# Supplementary material for: Effect of Hyperbaric Oxygen and Inflammation on Human Gingival Mesenchymal Stem/Progenitor Cells
Source: Cells. 2023 Oct 18;12(20):2479. doi: 10.3390/cells12202479 (PMC10605813; doi:10.3390/cells12202479)
Supplement: Supplementary file 1 [file cells-12-02479-s001.zip › cells-2620367-supplementary.pdf]

Supplements

Suppl. Table S1. Tissue donors.

Patient	Gender	Age	Known Illnesses	Smoking
1	♂	37	None	No
2	♂	47	None	No
3	♂	23	None	No
4	♂	38	None	No
5	♀	24	None	No

Suppl. Table S2. Additional contrasts in DEA analyses.

Effect of	Adjusted for confounder	N DE Genes	Top Genes	LFC (FDR P-Value)	Top KEGG Pathway
(1) Inflammation medium compared to standard medium without HBO treatment 24h	donor	760	CLDN	4.63 (4.77e-09)	Cytokine-Cytokine receptor Interaction
			SERPINB2	2.95 (3.53e-08)	
			TNFAIP3	3.64 (3.74e-08)	
(2) Inflammation medium compared to standard medium without HBO treatment 72h	donor	741	COLEC10	-2.46 (1.33e-05)	Cytokine-Cytokine receptor Interaction
			SLC7A2	2.73 (1.88e-05)	
			CD74	2.43 (2.17e-05)	

DE: differentially expressed; LFC: Log-Fold Change; FDR: False Discovery rate.

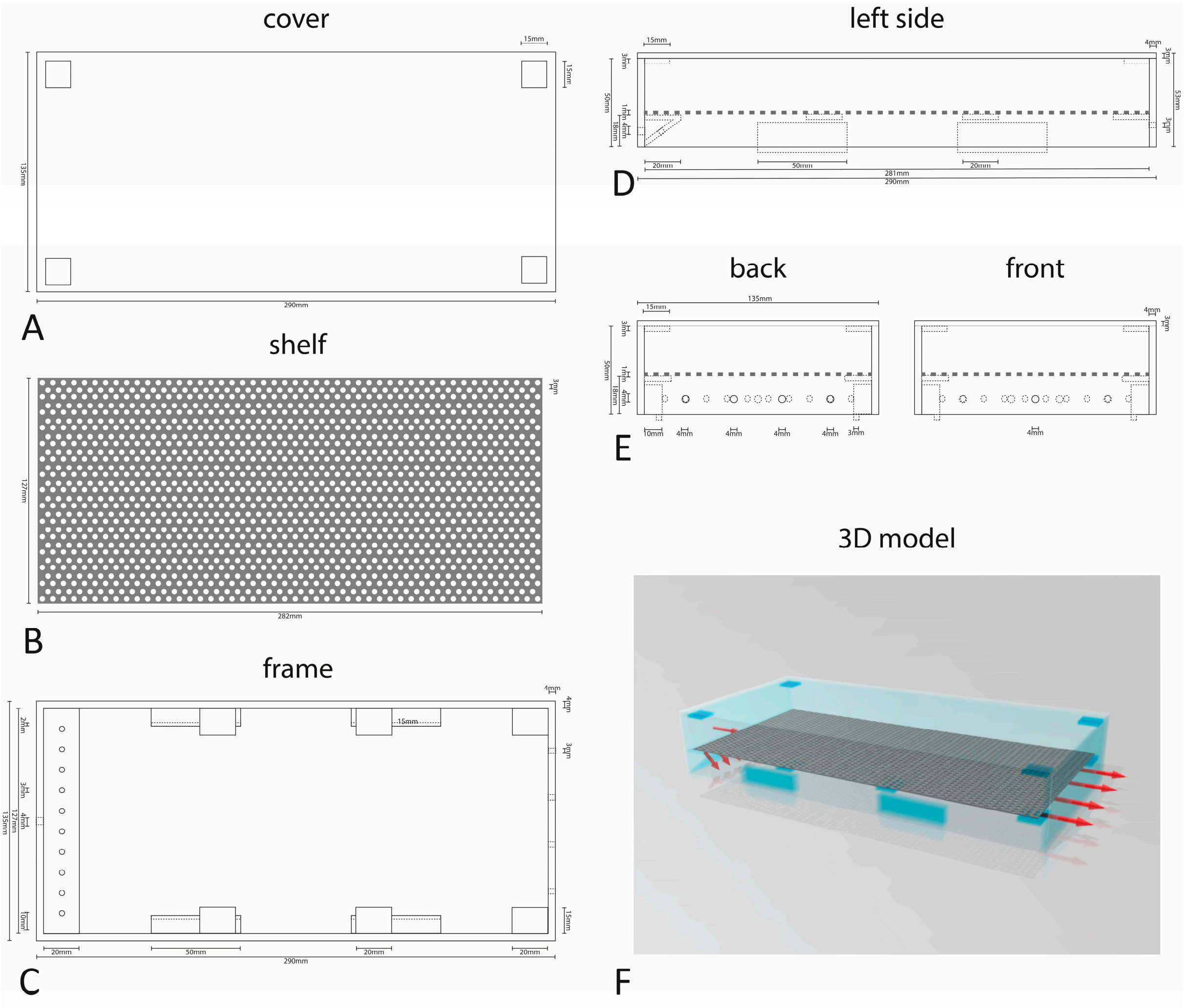

Suppl. Figure S1. Experimental set-up in 6 to 24 well plate HBO stimulation. High oxygen levels were achieved using an individually designed gas chamber. Cover (A) and frame (C) were made of acrylic glass; the shelf was cut out of a perforated metal plate. Assembled (D-E) Wells and petridishes were placed on the shelf and the frame was lowered into 37°C water, initiating constant stream of oxygen (F).

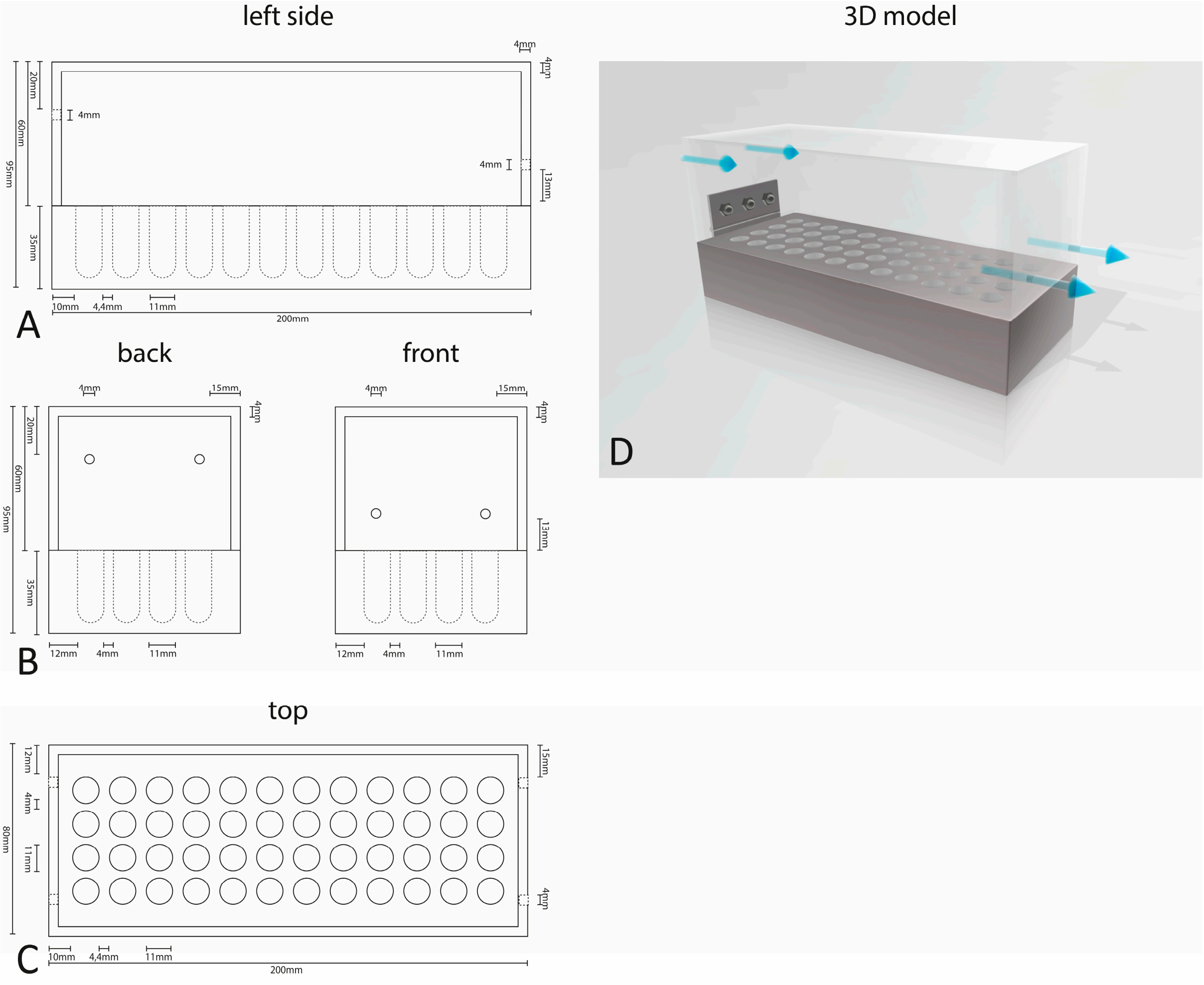

Suppl. Figure S2. Experimental set-up in 1.5ml Eppendorf Tube HBO stimulation. Oxygen supplement in chondrogenic differentiation required a specialized kind of chamber. The bottom was milled out of a bar of aluminum, the top, again, was made of acrylic glass (A-C). The bottom was placed in 37°C water during HBO stimulation (D).
